# Traffic Light Labels and Dietary Behavior Change: A Randomized Clinical Trial

**DOI:** 10.1001/jamanetworkopen.2025.10894

**Published:** 2025-05-19

**Authors:** Hongwei Liu, Zihan Hu, Qi Song, Jinji Xu, Shupeng Mai, Zhenni Zhu

**Affiliations:** 1 School of Public Health, Fudan University, Shanghai, China; 2 Division of Health Risk Factors Monitoring and Control, Shanghai Municipal Center for Disease Control and Prevention, Shanghai, China

## Abstract

**Importance:**

Evidence from everyday dining situations regarding the effects of traffic light labels (TLLs) on dietary improvement remains inconsistent.

**Objective:**

To evaluate the effects of TLLs on dietary consumption and choices in cafeteria settings.

**Design, Setting, and Participants:**

This 2-arm, parallel randomized clinical trial was conducted at a company staff cafeteria in Shanghai, China, from September to December 2022. Of 153 adult participants, 76 were randomly assigned to the intervention group and 77 to the control group. Data analysis was conducted from July to October 2024.

**Intervention:**

The intervention group gained access to TLLs offering a comprehensive rating of added sugar, fat, and sodium for each dish on the lunch menu, while the control group did not.

**Main Outcomes and Measures:**

Primary outcomes were lunch intake of added sugar, fat, and sodium. Secondary outcomes included the mean traffic light score (calculated based on the number of dishes consumed, with higher scores indicating worse overall dietary choices) and number of green-coded (reaching dietary recommendations), yellow-coded (between the recommendation and mean intake of the Chinese population), and red-coded (above the upper limit of intake) dishes. The primary and secondary outcomes were automatically calculated based on the precollected recipe dataset of the cafeteria and the Chinese food composition database when participants ordered meals using an applet during weekday lunchtime. Weekly median values of these outcomes were used for analyses, which were conducted for the intention-to-treat population.

**Results:**

Among 153 participants, the mean (SD) age was 32.7 (7.5) years, and 97 (63.4%) were female. At week 12, compared with the control group, the intervention group demonstrated no statistically significant decrease in dietary consumption of added sugar (mean difference, −0.15 [95% CI, −0.75 to 0.46] g), fat (mean difference, −1.54 [95% CI, −6.13 to 3.05] g), or sodium (mean difference, −116.12 [95% CI, −454.78 to 222.54] mg). Similarly, no statistically significant differences were observed in dietary choices based on the mean traffic light score (mean difference, −0.05 [95% CI, −0.12 to 0.03]) or the number of green-coded (odds ratio [OR], 1.15 [95% CI, 0.99-1.32]), yellow-coded (OR, 1.04 [95% CI, 0.90-1.20]), and red-coded (OR, 0.84 [95% CI, 0.57-1.23]) dishes.

**Conclusions and Relevance:**

In this randomized clinical trial, TLLs indicating added sugar, fat, and sodium ratings on menus failed to improve dietary consumption and choices in a company cafeteria setting. This finding suggests that TLLs on menus may not effectively promote dietary improvement in this setting.

**Trial Registration:**

Chinese Clinical Trial Registry Identifier: ChiCTR2100051771

## Key Points

**Question:**

In everyday cafeteria settings, do traffic light labels improve dietary consumption and choices?

**Findings:**

In this randomized clinical trial including 153 adults, those who gained access to traffic light labels indicating ratings of added sugar, fat, and sodium in dishes offered at a company cafeteria during lunch did not show statistically significant improvement in dietary consumption and choices throughout the 3-month study period.

**Meaning:**

These findings suggest that traffic light labels of added sugar, fat, and sodium on menus may not effectively promote dietary improvements in everyday cafeteria settings.

## Teaser

This randomized clinical trial evaluates whether gaining access to traffic light labels providing ratings of fat, sugar, and sodium content while ordering lunch at a cafeteria improves dietary choices and consumption among adults.

## Introduction

The dramatic development of the catering industry, coupled with fast-paced lifestyles, has made dining out more prevalent in China.^1^ This shift has substantially affected diets, resulting in higher intake of added sugars, fat, and sodium, which is a major contributing factor to increased risk and prevalence of obesity and noncommunicable diseases.^2,3^ Providing key nutritional information of foods in a visible format, such as traffic light labels (TLLs) and warning labels, is recognized as a cost-effective strategy to promote healthful diets.^4,5^

Compared with other types of nutrition labeling systems, TLLs are deemed to perform better in promoting the choice and consumption of healthier foods.^6,7^ However, some studies indicate that while TLLs perform well in improving perception and behavioral intentions, evidence from everyday practices is mixed and limited regarding the effectiveness of TLLs for eventually improving dietary consumption and choices.^8,9,10^ Some evidence indicates that replicating the intervention effects from randomized clinical trials (RCTs) in everyday practice is quite difficult.^7,8,11^ For example, a 2016 field experiment revealed that the effect sizes observed in the study were 17 times smaller than those reported in comparable laboratory studies conducted under rigorously controlled conditions.^12^

Meanwhile, the effects of TLLs are significantly influenced by study participants’ demographic characteristics, nutrition literacy, and cooking culture.^6,13^ This may be one of the main reasons for inconsistent results and puts more emphasis on studying the effects of TLLs outside of controlled environments in a particular culture. The objective of the present study was to evaluate the effect of TLLs on enabling timely healthier dietary consumption and choices in an everyday situation.

## Methods

### Setting and Study Design

Briefly, the study was conducted as a 2-arm, parallel RCT to examine the effect of a dietary intervention provided by an applet. We conducted the study in a company staff cafeteria in Shanghai, China. During the study, participants were free to follow their daily routine and consume meals of their own choice. More details are provided in previous studies^14,15^ and in the Trial Protocol in Supplement 1. This study followed the Consolidated Standards of Reporting Trials (CONSORT) reporting guideline and was approved by the Ethics Committee of Shanghai Municipal Center for Disease Control and Prevention. Written informed consent was obtained from all participants.

### Participants

Recruitment commenced September 2022, with the study running from September to December 2022. Inclusion and exclusion criteria are given in the Trial Protocol provided in Supplement 1. The planned sample size per group was 70 to detect an assumed intervention effect size of 0.25 in animal to plant food ratios across 4 months. Statistical power was set at 90%, with a significance level of 5%, using an SD in the animal to plant food ratio of 0.41 from the Shanghai Diet and Health Survey^16^ and considering a 15% dropout rate.

### Randomization and Blinding

Participants were randomized to either the intervention or control group in a 1:1 ratio. The trial was conducted as a double-blind study, with field investigators and participants blinded to the study groups.

### Run-In Phase and Baseline Assessment

Participants underwent a 1-week run-in period to learn to use the applet for ordering dishes and entering leftover proportions. Both groups were unable to access the applet’s dietary nutrition evaluation functionality during this phase.

### Interventions

Following the 1-week run-in period, the participants in the intervention group gained access to the dietary nutrition evaluation functionality, including premeal traffic light illustrations of dishes (eFigure 1B in Supplement 2) and postmeal nutrition reports (eFigure 1C in Supplement 2). The explanations for premeal and postmeal interventions were provided via pop-up windows in the applet’s interface (eFigure 2 in Supplement 2). The control group did not have this access. Both groups were continually instructed to use the applet for ordering dishes during weekday lunch. In the cafeteria, each dish was served in equal-weight single portions for diners (eFigure 1A in Supplement 2). Through integrating the precollected recipe dataset of all the dishes offered in the cafeteria with the publicly accessible Chinese food composition database at the applet’s back end, the real-time realization and visualization of the applet’s dietary nutrition evaluation functionality were achieved as the participants ordered dishes via the applet in the cafeteria.^15,17^ Both groups were free to choose and consume their meals.

### Premeal and Postmeal Interventions

Using the food-ordering interface, participants could browse and choose dishes. For the intervention group, each dish was labeled with a colored dot next to its name representing the levels of added sugar, fat, and sodium in the dish (eFigure 1B in Supplement 2). The dots were color-coded using a traffic light system (green, reaching dietary recommendations^18^; yellow, between the recommendation and mean intake of the Chinese population^19^; and red, above the upper limit of intake). Nutrient cutoffs for the color codes and calculation rules are provided in eTable 1 and eMethods 1 in Supplement 2. After ordering dishes and entering the leftover proportion of each meal, participants in the intervention group received a real-time nutrition report taking into account the leftover proportion of each dish^20,21,22^ (eFigure 1C in Supplement 2).

### Measures and Follow-Up

#### Baseline Assessment

Data on age (years), sex (male or female), smoking status (nonsmoker, exsmoker, or current smoker), alcohol consumption (lifetime abstainer, nonheavy consumer, or heavy consumer), physical activity (low, moderate, or vigorous), intentional physical exercise (yes or no), body mass index (BMI; calculated as weight in kilograms divided by height in meters squared and categorized as underweight [<18.5], normal weight [18.5-23.9], overweight [24.0-27.9], or obesity [≥28.0]), nutrition literacy (scale range, 0-5, with higher scores denoting greater nutrition literacy), and demand for dietary guidance (scale range, 0-3, with higher scores denoting greater demand for dietary guidance) were collected through baseline survey. Details on these variables are provided in eTable 2 in Supplement 2.

#### Outcomes

The primary outcome was lunch dietary intake, including intakes of added sugar, fat, and sodium. The secondary outcomes included lunch mean traffic light score and number of green-coded, yellow-coded, and red-coded dishes. The mean traffic light score was calculated based on the number of dishes consumed by the participants, with higher scores indicating worse overall dietary choices (eMethods 2 in Supplement 2).

The primary and secondary outcomes were recorded by the applet at baseline (1-week run-in period) and the follow-up period. In the cafeteria, all dishes were prepared using fixed recipes and portioned into uniform single servings for diners. The recipe dataset containing the measured weights of food components and condiments for each dish, along with the Chinese food composition database, was uploaded to the applet’s back end.^15,17^ On weekdays, when participants chose to have lunch at the staff cafeteria, participants were instructed to use the applet to order dishes in the food-ordering interface and subsequently to enter the proportions of leftovers for each dish after meals. Once participants finished their meals and entered the proportions of leftovers via the applet, a detailed dietary report, including the primary and secondary outcomes, was automatically computed and recorded.

To account for fluctuations in dietary intakes, weekly median intakes were calculated and used to minimize the potential impact of extreme values of dietary intake on the analysis. For the number of different color-coded dishes consumed, the median of each color-coded dish per week was used, and 0.5 (ie, half-dish increments) was used as the unit of analysis.

### Statistical Analysis

The baseline characteristics of the participants are presented as means (SDs) for normally distributed continuous variables, medians (IQRs) for nonnormally distributed continuous variables, and frequencies (percentages) for categorical variables. All analyses were conducted for the intention-to-treat population. We also evaluated the comparability of demographic characteristics between the included and excluded participants. Multiple imputation using chained equations was conducted to impute missing covariate data, generating 5 imputed datasets for subsequent analyses. To evaluate potential bias introduced by the cafeteria meal service, we conducted the Mann-Kendall trend test to analyze the temporal trend in the mean traffic light score of the daily lunchtime menu throughout the study period.

Linear mixed models were conducted to analyze the effects of TLLs on the primary outcome and the mean traffic light score, and Poisson regression mixed models were used to assess the effects on the number of dishes over time.^23^ Time, group (intervention and control), and a 2-way interaction term between time and group were treated as fixed factors, with participant-level random intercepts and slopes to account for repeated measures. Between-group differences at week 12 were reported based on the models. Model 1 adjusted for the baseline value of the mean traffic light score consumed and enrollment sequence (September, October, and November). Model 2 included additional adjustments for age, sex, smoking status, alcohol consumption, physical activity level, intentional physical exercise, BMI, nutrition literacy, and demand for dietary guidance. A series of stratified analyses were performed by sex, BMI, nutrition literacy, and demand for dietary guidance to evaluate the consistency and robustness of the results.

All analyses were conducted from July to October 2024 using R, version 4.2.3 (R Project for Statistical Computing). The nlme package was used for linear mixed models, the lmerTest package for Poisson regression mixed models, and the mice package for multiple imputation. Statistical significance was defined as a 2-tailed *P* < .05.

## Results

### Participant Characteristics

**Figure 1.**
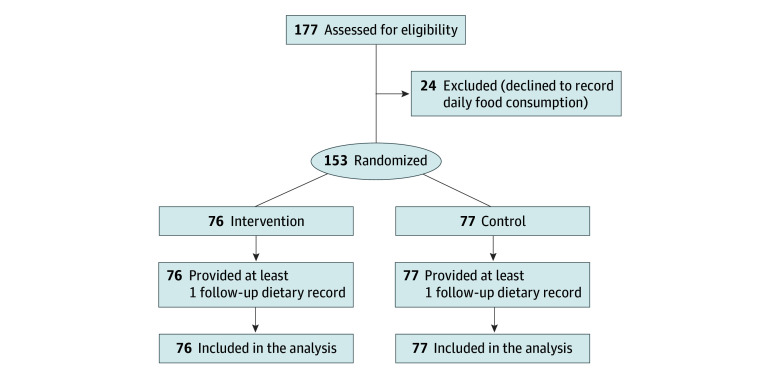
Study Flowchart

A total of 153 of 177 enrolled participants (97 [63.4%] women and 56 [36.6%] men; mean [SD] age, 32.7 [7.5] years) were randomly assigned to either the intervention (n = 76) or control group (n = 77) (Table 1 and Figure 1). During the study period, all participants submitted at least 1 dietary record, amounting to 4138 records. As shown in eTable 3 in Supplement 2, the excluded participants had a higher proportion with overweight and who were male, smoked, and consumed alcohol compared with the included participants.

**Table 1.** Baseline Characteristics of the Participants

Characteristic	Participants, No. (%)		
	All (N = 153)	Control (n = 77)	Intervention (n = 76)
Age, mean (SD), y	32.7 (7.5)	32.7 (7.3)	32.6 (7.8)
Sex			
Female	97 (63.4)	51 (66.2)	46 (60.5)
Male	56 (36.6)	26 (33.8)	30 (39.5)
Smoking status			
Nonsmoker	136 (88.9)	69 (89.6)	67 (88.2)
Exsmoker	11 (7.2)	5 (6.5)	6 (7.9)
Current Smoker	6 (3.9)	3 (3.9)	3 (3.9)
Alcohol consumption^a^			
Lifetime abstainer	67 (43.8)	34 (44.2)	33 (43.4)
Nonheavy consumption	68 (44.4)	35 (45.4)	33 (43.4)
Heavy consumption	18 (11.8)	8 (10.4)	10 (13.2)
Physical activity			
Low	134 (87.6)	70 (90.9)	64 (84.2)
Moderate	18 (11.8)	7 (9.1)	11 (14.5)
Vigorous	1 (0.7)	0	1 (1.3)
Intentional physical exercise			
No	118 (77.1)	58 (75.3)	60 (78.9)
Yes	35 (22.9)	19 (24.7)	16 (21.1)
Enrollment period			
September	23 (15.0)	10 (13.0)	13 (17.1)
October	86 (56.2)	42 (54.5)	44 (57.9)
November	44 (28.8)	25 (32.5)	19 (25.0)
BMI^b^			
Underweight	6 (3.9)	4 (5.2)	2 (2.6)
Normal	96 (62.7)	55 (71.4)	41 (53.9)
Overweight	36 (23.5)	14 (18.2)	22 (28.9)
Obesity	15 (9.8)	4 (5.2)	11 (14.5)
Traffic light score, mean (SD)^c^	1.7 (0.3)	1.7 (0.3)	1.7 (0.3)
No. of dishes consumed, median (IQR)			
Green-coded	2.0 (1.0-2.0)	2.0 (1.0-2.0)	1.0 (1.0-2.0)
Yellow-coded	2.0 (1.0-2.0)	2.0 (1.0-2.0)	2.0 (1.0-2.0)
Red-coded	0.0 (0.0-1.0)	0.0 (0.0-1.0)	0.0 (0.0-1.0)
Dietary intake, mean (SD)			
Added sugar, g/meal	1.7 (1.9)	1.5 (1.6)	2.0 (2.2)
Fat, g/meal	36.4 (22.6)	37.4 (24.1)	35.3 (21.1)
Sodium, mg/meal	2129.6 (1159.2)	2059.0 (1040.6)	2201.1 (1271.1)
Demand for dietary guidance, mean (SD)^d^	1.9 (0.7)	1.9 (0.8)	2.0 (0.7)
Missing	5 (3.3)	4 (5.2)	1 (1.3)
Nutrition literacy, mean (SD)^e^	3.3 (1.0)	3.3 (1.0)	3.3 (1.0)
Missing	5 (3.3)	4 (5.2)	1 (1.3)

Abbreviation: BMI, body mass index (calculated as weight in kilograms divided by height in meters squared).

^a^ Alcohol consumption was assessed through self-reported frequency of alcohol intake during the baseline questionnaire survey. Participants were categorized as lifetime abstainers (never having consumed alcohol), nonheavy consumption (alcohol consumption 1-6 times per year), and heavy consumption (alcohol consumption >6 times per year).

^b^ BMI was categorized as underweight, <18.5; normal weight, 18.5-23.9; overweight, 24.0-27.9; and obesity ≥28.0.

^c^ Traffic light scores ranged from 1 to 3, with higher scores indicating worse overall dietary choices.

^d^ Demand for dietary guidance scale ranged from 0 to 3, with higher scores denoting greater demand for dietary guidance.

^e^ Nutrition literacy scale ranged from 0 to 5, with higher scores denoting greater nutrition literacy.

### Cafeteria Meal Supply

During the study period, the median (IQR) number of dishes served for daily lunch in the cafeteria was 14 (14-14), with 6 (5-6) green-coded, 7 (6-8) yellow-coded, and 2 (1-2) red-coded dishes (eTable 4 in Supplement 2). In total, the cafeteria provided 241 dishes, including 78 green-coded, 121 yellow-coded, and 42 red-coded dishes. The results of the Mann-Kendall trend analysis shown in eFigure 3 in Supplement 2 indicated that there was no significant trend over time in the mean traffic light scores of the daily lunch menu.

### Effects of the Intervention on Outcomes Throughout the Study Period

**Figure 2.**
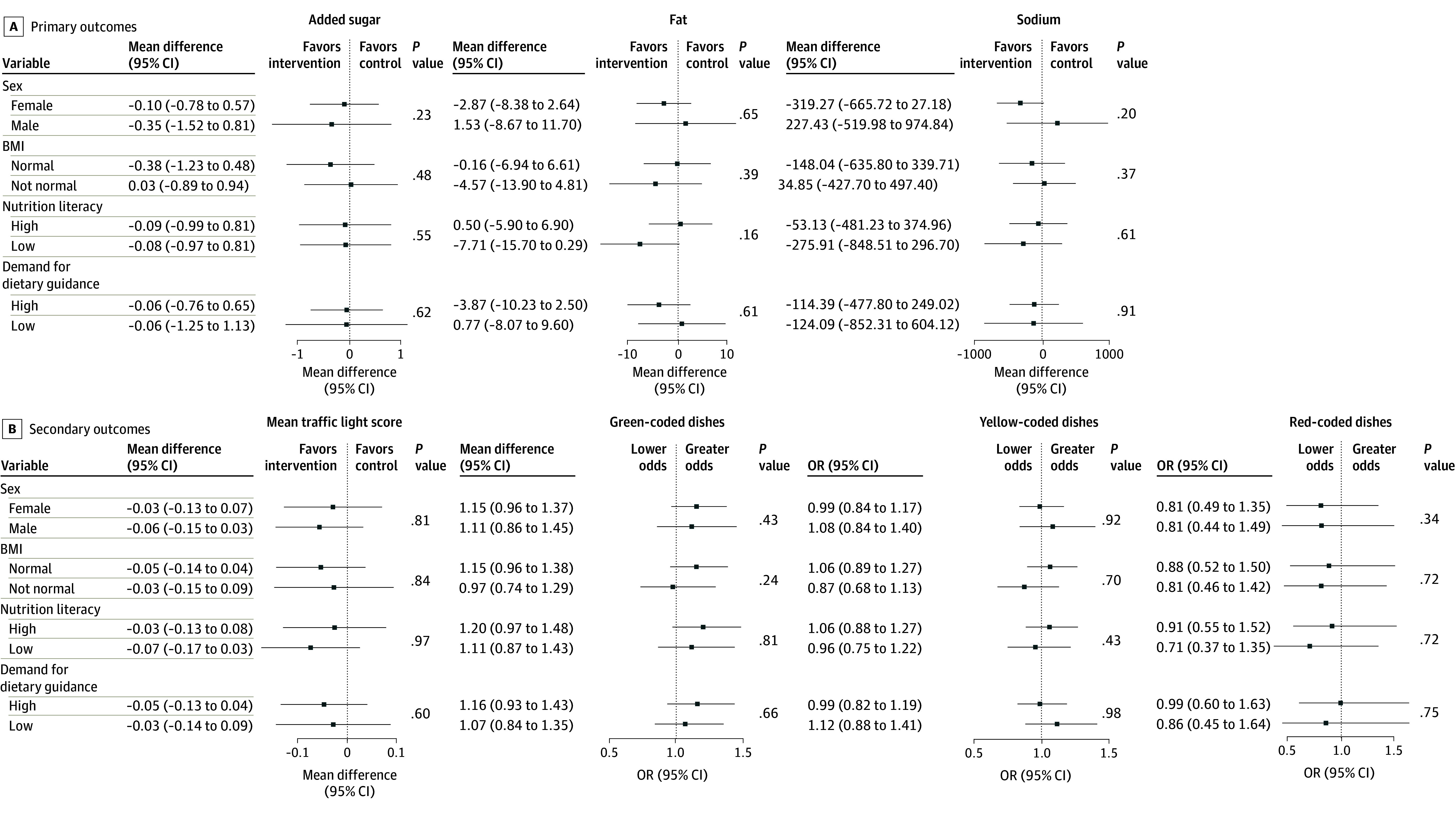
Effects of the Intervention on Primary and Secondary Outcomes Across Various Subgroups at Week 12 in Model 2 Body mass index (BMI; calculated as weight in kilograms divided by height in meters squared) was reclassified to normal (18.5-23.9) or not normal (underweight [<18.5], overweight [24.0-27.9], and obesity [≥28.0]). Both nutrition literacy and demand for dietary guidance were separated into low (scores below the median) or high (scores at or above the median) categories, with higher scores representing greater nutrition literacy or greater demand for dietary guidance, respectively. OR represents odds ratio.

Table 2 gives the between-group differences of the primary and secondary outcomes at week 12, with the overall between-group differences for the outcomes displayed in eTable 5 in Supplement 2. At week 12, compared with the control group, the intervention group did not exhibit statistically significant changes in intake of added sugar (mean difference, −0.15 [95% CI, −0.75 to 0.46] g), fat (mean difference, −1.54 [95% CI, −6.13 to 3.05] g), and sodium (mean difference, −116.12 [95% CI, −454.78 to 222.54] mg). In addition, no statistically significant differences were observed in mean traffic light scores (mean difference, −0.05 [95% CI, −0.12 to 0.03]) or the number of green-coded (odds ratio [OR], 1.15 [95% CI, 0.99-1.32]), yellow-coded (OR, 1.04 [95% CI, 0.90-1.20]), and red-coded (OR, 0.84 [95% CI, 0.57-1.23]) dishes consumed at lunch between the intervention and control group. Stratified analyses found that the effect of the intervention on the primary and secondary outcomes did not significantly differ by sex, BMI, nutrition literacy, or demand for dietary guidance (Figure 2 and eFigure 4 in Supplement 2).

**Table 2.** Effect of the Intervention on Primary and Secondary Outcomes at Week 12

Outcome	Model 1^a^				Model 2^b^		
	Mean difference (95% CI)	OR (95% CI)	*P* value	Mean difference (95% CI)		OR (95% CI)	*P* value
Dietary intake							
Added sugar, g/meal	−0.15 (−0.77 to 0.47)	NA	.64	−0.15 (−0.75 to 0.46)		NA	.63
Fat, g/meal	−1.26 (−6.06 to 3.54)	NA	.61	−1.54 (−6.13 to 3.05)		NA	.51
Sodium, mg/meal	−123.24 (−477.63 to 231.15)	NA	.50	−116.12 (−454.78 to 222.54)		NA	.50
Mean traffic light score^c^	−0.05 (−0.12 to 0.02)	NA	.15	−0.05 (−0.12 to 0.03)		NA	.21
No. of dishes							
Green-coded	NA	1.12 (0.96 to 1.30)	.15	NA		1.15 (0.99 to 1.32)	.06
Yellow-coded	NA	1.02 (0.88 to 1.18)	.78	NA		1.04 (0.90 to 1.20)	.63
Red-coded	NA	0.83 (0.56 to 1.23)	.35	NA		0.84 (0.57 to 1.23)	.36

Abbreviations: NA, not applicable; OR, odds ratio.

^a^ Model 1 adjusted for the baseline value of mean traffic light score consumed and enrollment sequence.

^b^ Model 2 adjusted for the baseline value of mean traffic light score consumed, enrollment sequence, age, sex, smoking status, alcohol consumption, physical activity level, intentional physical exercise, body mass index, nutrition literacy, and demand for dietary guidance.

^c^ Traffic light scores ranged from 1 to 3, with higher scores indicating worse overall dietary choices.

## Discussion

This RCT conducted in a company staff cafeteria determined that TLLs failed to improve the participants’ dietary consumption and choices during the study period. TLLs are considered a cost-effective population-level intervention to promote healthier dietary behaviors. However, evidence regarding the effectiveness of TLLs in sustainably improving dietary consumption and choices remains inconclusive, particularly in community-based settings in which contextual factors (eg, food accessibility, cultural preferences) may significantly influence intervention effects.

In the current study, compared with the control group, the intervention group did not reduce the consumption of added sugar, fat, and sodium over time during the study period, consistent with studies conducted in other settings.^8,24,25^ This finding was further supported by the results of the secondary outcomes indicating that the dietary choices of the intervention group did not become healthier, including overall dietary choices and specific foods with different color-coded labels. Recent studies have indicated that while TLLs have demonstrated moderate effectiveness on outcomes such as consumer perception and behavioral intentions, they generally underperform in transforming actual consumption behaviors. For example, an RCT conducted in 6 college cafeterias found that although 75% of the participants in the intervention group reported that TLLs were useful and most participants wanted the intervention to continue, TLLs did not significantly change portions of unhealthy or healthy foods served in intervention vs control sites.^8^ TLLs use color scales to classify foods from green (most healthy) to red (least healthy) by scoring the level of each target nutrient, such as added sugar, fat, and sodium, which may confuse consumers by sending mixed messages.^7,26^ This conclusion was supported by a review assessing nutrition labeling practices in community settings, which determined that using simple warning symbols, instead of such mixed labels, was more conducive to enabling consumers to interpret nutrition information quickly and accurately.^27^ However, these results are inconsistent with some studies that have reported significant improvement in dietary quality associated with the use of TLLs.^28,29^ This inconsistency across studies may partly result from differences in the study design.^24,30^ Compared with laboratory and other controlled settings, the effect size observed in open community settings is smaller and even toward the null.^12,27^ Previous studies also observed that the effects of TLLs gradually diminished and became null over time.^8,28^ Thus, compared with studies with a short follow-up period, studies with long follow-up periods are more likely to find no significant associations of TLLs with dietary consumption and choices. The reasons for this phenomenon may be 2-fold. First, less attention was given to labels over time, referred to as label fatigue.^8^ Studies have observed that compared with label systems that grade foods on a negative to positive scale (eg, TLLs), simple and negative warning labels are easier and quicker for consumers to notice and sustainably use in practice.^27,31,32^ Second, other barriers to long-term dietary improvements include food taste, food accessibility, and nutrition publicity.^27,28,33,34^

In the present study, TLLs were passively delivered as informational aids without accompanying dietary restrictions, prescriptive guidance, or educational campaigns. Participants autonomously selected dishes, mirroring exposure to nutrition labels in settings lacking systematic public health support.^35^ Further studies are warranted to evaluate the effects of integrating complementary educational or promotional strategies with TLLs for long-term dietary improvements.

To our knowledge, this study is the first RCT conducted to evaluate the effect of TLLs in a cafeteria in China, providing valuable insights for developing an effective nutrition labeling system under cafeteria settings. The mean traffic light score of the daily lunch menu demonstrated no significant trend over time after the study began, and the distribution and quantities of green-coded, yellow-coded, and red-coded dishes in the daily and total menus were reasonable. Thus, the findings may not be attributable to the dining environment. Dietary reports were automatically recorded and calculated in real-time, which would avoid systematic measurement errors and recall bias and enhance the reliability and validity of the results.

### Limitations

This study has several limitations. First, self-reported leftover proportions may result in discrepancies from actual leftover proportions per lunch. Second, this study provided the intervention and analyzed dietary consumption and choices solely during weekday lunches. Participants’ dietary consumption and choices during breakfast, dinner, and on weekends were not monitored, limiting our findings to only a partial reflection of the intervention effect. Third, despite our adjustment for a broad range of confounders, residual confounding may persist due to unmeasured covariates (eg, educational level and socioeconomic status). Fourth, the study was conducted in a cafeteria setting with limited variety in the daily menu, which may constrain external validity when generalizing findings to other settings with diverse food environments. In addition, mixed sauces, including added sugar, cooking oil, and salt, were used in certain dishes, which may lead to underestimation of actual intake of added sugar. However, we collected and analyzed myriad nutrients from these mixed sauces in detail, especially fat and sodium.

## Conclusions

In this RCT conducted in a staff cafeteria in China, TLLs offering a comprehensive rating of added sugar, fat, and sodium for each dish on the lunch menu failed to decrease the consumption of added sugars, fats, and sodium or to enhance dietary choices throughout the study period. These findings suggest that TLLs on menus may not effectively promote dietary improvements.

## Notes

Supplement 1.
Trial Protocol and Statistical Analysis Plan

Supplement 2. **eFigure 1.** The demonstration of the study canteen and the applet interfaces **eFigure 2.** The “Direction” of the applet interfaces **eTable 1.** Cutoffs of nutrients for “traffic light” classification on dishes **eMethods 1.** Calculation rules for “traffic light” classification on dishes **eTable 2.** The definition of covariates **eMethods 2.** Calculation rules for the average traffic light score **eTable 3.** Comparison of baseline characteristics between the participants included and excluded **eTable 4.** The distribution and quantity of green-coded, yellow-coded, and red-coded dishes in the daily and total menus **eFigure 3.** The temporal trend in the average traffic light score of the daily lunchtime menu **eTable 5.** Effects of the intervention on weekly changes of primary and secondary outcome **eFigure 4.** Effects of the intervention on primary and secondary outcomes across various subgroups at week 12 in model 1

Supplement 3.
Data Sharing Statement
