# Supplementary material for: Traffic Light Labels and Dietary Behavior Change: A Randomized Clinical Trial
Source: JAMA Netw Open. 2025 May 19;8(5):e2510894. doi: 10.1001/jamanetworkopen.2025.10894 (PMC12090027; doi:10.1001/jamanetworkopen.2025.10894)
Supplement: Supplement 1. — Trial Protocol and Statistical Analysis Plan [file jamanetwopen-e2510894-s001.pdf]

Effects of Traffic Light Labels on Improving Dietary Choices and Consumption: A Randomized Controlled Trial under Unrestricted Real-World Scenarios

Final study protocol

Background

Suboptimal dietary pattern is a major contributing factor to the growing incidence of non-communicable diseases (NCDs) in China, which is characterized by a high intake of fat and sodium and a low intake of vegetables and fruits [1,2]. The rapid modernization of the restaurant and packaged food industry has aggravated unhealthy dietary pattern [3]. China has set a nationwide target to reduce the intake of dietary oil, salt, and sugar, as well as promoting nutrition education to help the public adopt healthier eating habits [4]. However, recent data reveals that in comparison to the number of registered dietitians (RDs) and registered dietetic technicians (DTRs) in developed countries, China has a much lower ratio, with only 67 RDs/DTRs per 10 million people, which is far from meeting the massive needs of nutrition guidance [5].

PN (personalized nutrition) is a field that utilizes human individuality to develop nutrition approaches that prevent, manage, and treat diseases and optimize health [6]. Previous studies have suggested that smartphones with built-in AI (artificial intelligence)-based algorithms are cost-effective, flexible, visually appealing and engaging, which could approach PN guidance and therapeutics provided by real healthcare professionals [7,8].

Substantial evidence indicates that randomized controlled trials (RCTs) hardly shift into real-world practice [9]. In most long-term weight loss studies in humans, favorable results were achieved during the intervention but hard to keep after returning to normal life [10]. In contrast, real world studies allow participants to follow their daily routines and enjoy real-life meals on wills, which might facilitate a trial becoming a real-world practice [11].

We have developed a PN approach with an AI-based smartphone applet, which aims to help provide the information of dish nutrition evaluation and PN evaluation after meal consumption for the mass of people under the circumstance of dietitian

shortage in China. This study is to identify the effectiveness and feasibility of this novel approach in a parallel, randomized, controlled trial in real world scenario.

Methods

Setting and study design

This trial is a two-arm parallel, randomized, controlled study in real-world situations. This smartphone applet with built-in AI-based algorithm only takes effect where dishes prepared by recipes with quantity (in China, same dish prepared at different home are different in cooking but dishes in central kitchen or food factory are prepared strictly following standard cooking procedure [12]). Participants are enrolled sequentially until the sample size reaches requirement. This study will be carried out in a pilot company employing 3000 people and food is prepared by the central kitchen at staff canteen. All participants will choose and consume meals on their own wills during the study. Weight and blood pressure measuring tools placed at the canteen can be available to participants; therefore, they can conveniently measure and record anthropometric indicators through the applet.

Phase I. The employees will be recruited to dine in the canteen for 3 months, assigned to the intervention or control group randomly. Both groups will be asked to use the applet to record their lunch each weekday during the study. After 1 week of run-in period, the intervention group will be able to access the information of dish nutrition evaluation and PN evaluation after meal consumption, while the control group will not. Both groups will be followed up by researchers on the same time schedules for the outcome measurements.

Phase II. Full-functioned applet will be available for use by all the diners (about 800) for another 1 year. Who use the applet at least 2 days per week will be regarded as the intervention group while the others will be the control group. During this phase, metabolic indicators from the annual physical examination will be provided by the company.

Figure 1 outlines the study design and Table 1 presents the visits and data

collection schedule.

Figure 1. Study design of the trial.

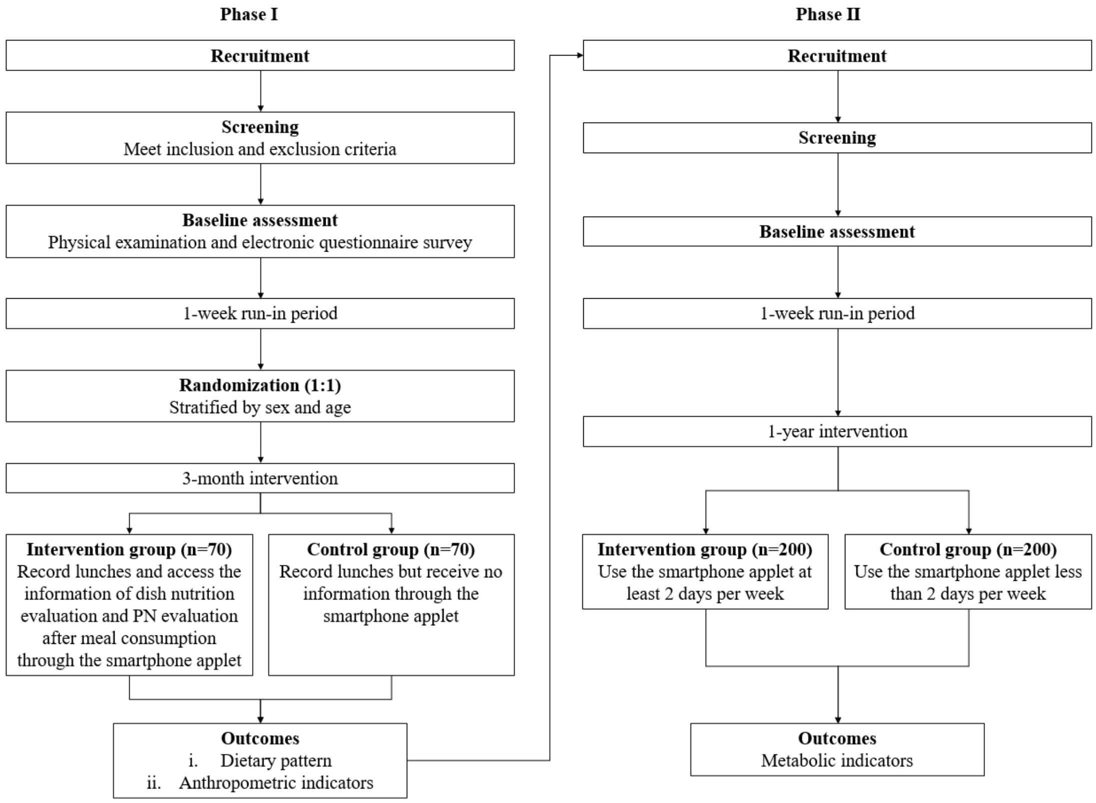

Table 1. Visits and data collection schedule

	Phase I (3 months)			Phase II (1 year)		
	Screening	Run-in	Follow-up	Screening	Run-in	Follow-up
Informed consent	√			√		
Questionnaire		√	√		√	√
Dietary record		√	√		√	√
Anthropometric indicators		√	√		√	√
Metabolic indicators					√	√

Sample size

In Phase I, the planned sample size per group was determined as 70 to detect an assumed intervention effect size of 0.25 in animal/plant food ratio over 4 months. Statistical power was set at 90% with a significance level of 5%, using an SD in animal/plant food ratio of 0.41 from the Shanghai Diet and Health Survey [13], considering a 15% dropout rate. In Phase II, assuming that the prevalence of

metabolic syndrome after intervention is approximately 20% based on the available research evidence [14], we would need 200 participants in each group to have sufficient power to detect the given effect size.

Procedure

Inclusion and exclusion criteria

Men and women will be eligible to participate if they (1) are more than 18 years old, (2) are healthy in appearance, (3) promise to have lunch at the staff canteen during the study period, (4) agree to record food consumption of each meal on the applet. Exclusion criteria include: (1) planning to change physical activity habits during the course of study; (2) unable to follow a regular diet (e.g. on diet).

Recruitment and follow-up

Pilot. To establish the feasibility and acceptability of current protocol, we recruited 94 employees to perform a 3-week, single-arm preliminary pilot trial and users tested this applet (data collection from February 2022 to March 2022).

Phase I. Recruitment and physical examination are planned to conduct at regular intervals during 3 months. Researchers ask employees about participation in the study, collect written informed consent from participants, instruct them to use the applet as well as measure their anthropometric indicators in a standardized way. Thereafter, participants complete demographic questions (birthdate, sex and occupation) through the applet, as well as answer questions regarding their family history of NCDs, physical fitness, drug use, physical activity level (PAL), sleep, smoking status, alcohol consumption, dietary habits and nutrition service needs. The follow-up questionnaire will be repeated at the end of the phase I and questions about user experience of this applet will be added to it.

Phase II. The intervention will last for another 1 year. All the diners will access the full-functioned applet. Metabolic data will be collected from the annual physical examination by the company. Participants will complete the same electronic questionnaire at pre- and post-intervention.

Randomization and blinding

Following baseline measurements, the newly-enrolled participants will be randomized at a ratio of 1:1 to an intervention or control group. Randomization is stratified by sex and age. The trial is conducted as a double-blind study. The researchers are not blinded to allocation due to the nature of the intervention strategy, however the field investigators and participants will be blinded throughout the study, ensuring allocation concealment.

The intervention

The intervention is to provide AI-based dish nutrition evaluation and PN evaluation after meal consumption at the study canteen through the applet (Figure 2). The applet based on privacy-preserving computing platform has two interfaces: a phone-based client and a web-based data management system. For users, this applet is a WeChat mini-program which has ease of use, high acceptance and little memory [15]. Similar to Facebook, WeChat is a very popular social software in China. The mini-program relying on WeChat can achieve health functions and provide native app-like experiences without leaving WeChat interface. For researchers, the web-based system is used to facilitate central management including user registration, recipe preparation, menu administration and information storage. The built-in AI algorithm is applied in data operation and processing to support iterative calculation.

Figure 2. User interfaces of the AI-based PN management applet.

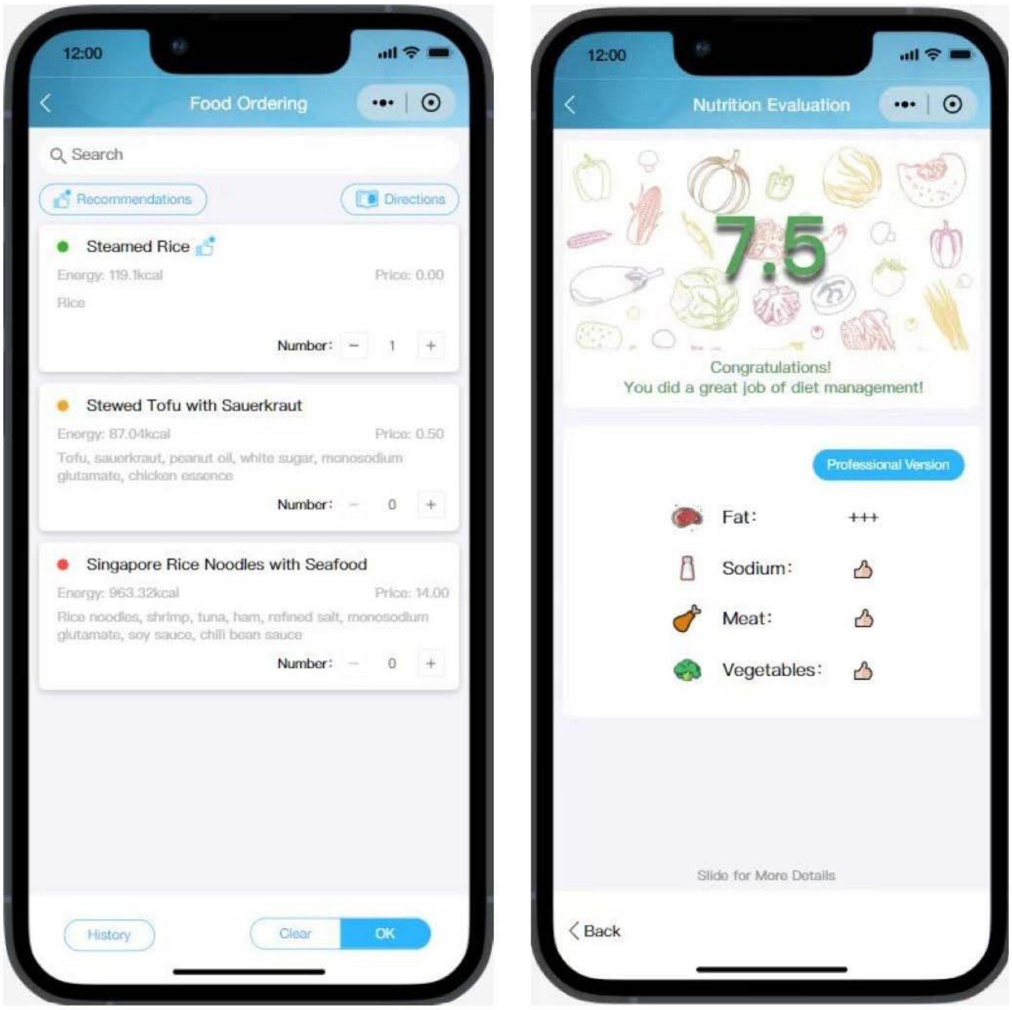

Screen 1 Screen 2

Note: Screen 1 demonstrates dish nutrition evaluation. Screen 2 shows PN evaluation after meal consumption.

Dish nutrition evaluation

Dish nutrition evaluation is used to determine whether the content of fat, sodium and sugar in dish is benefit for health.

Two independent datasets are used for our study. First is the Chinese food composition database [16], which is publicly available. This database includes nutritional values for over 1110 food items and corresponding food groups. Second, we create a recipe dataset of all the dishes supplied in this canteen. The recipe includes the raw weights of materials (including ingredients and condiments) and their edible proportion, as well as gross and single-portion cooked weights of each

dish. Weight measurements are conducted by field investigators with expertise in nutrition under the same standard criteria.

We construct a full dish/nutrition dataset by connecting the recipe dataset to Chinese food composition database. Thus, the food groups, energy and nutrients for each dish are automatically calculated using Algorithm 1. Food groups are classified as cereals & tubers (grains, potatoes and tubers), vegetables (excluding legumes), fruits (including citrus), livestock and poultry meat, eggs and products, seafood, dairy, nuts, soybeans and products, cooking oil, salt and sugar. Plant food stands for cereals & tubers, vegetables, fruits, soybeans and products. Animal food stands for livestock and poultry meat, seafood, eggs and products. Nutrients include protein, fat, carbohydrate, cholesterol, sodium, calcium, iron, zinc and vitamin C.

Algorithm 1. Calculation of food groups, energy and nutrients for each dish

$$dish = (food\ item[1], food\ item[2], \ldots, food\ item[m]) \quad ^{1}$$

$$Weight_{food\ group,dish} = \sum_{i \in dish} Raw\ Weight[i] \times Edible\ Proportion[i] \times \frac{Cooked\ Weight_{single-portion\ dish}}{Cooked\ Weight_{gross\ dish}} \quad ^{2}$$

$$Energy_{dish} = \sum_{i \in dish} Raw\ Weight[i] \times Edible\ Proportion[i] \times \frac{Energy[i]}{100} \times \frac{Cooked\ Weight_{single-portion\ dish}}{Cooked\ Weight_{gross\ dish}} \quad ^{3}$$

$$Nutrient_{dish} = \sum_{i \in dish} Raw\ Weight[i] \times Edible\ Proportion[i] \times \frac{Nutrient[i]}{100} \times \frac{Cooked\ Weight_{single-portion\ dish}}{Cooked\ Weight_{gross\ dish}} \quad ^{4}$$

[1] By matching two datasets, food item represents raw material (ingredient or condiment) in dish.

[2] Similar food items are merged into predetermined food groups.

[3] According to Chinese food composition database, energy[j] refers to content of energy in 100g edible portion of food item[j].

[4] According to Chinese food composition database, nutrient[j] refers to content of nutrient in 100g edible portion of food item[j].

The intervenors can browse and choose dishes on the ordering interface of the applet. Colored dots are displayed next to the dishes’ names by a “traffic light” approach to indicate whether the dishes are benefit for health. The judgement of three colors is based on the contents of fat, sodium and sugar (green = reaching the dietary

recommendations, yellow = between the recommendations and average intakes among Chinese population [17], red = above the upper limit of intakes). The dietary recommendations in this study are: no more than 8g fat, 500mg sodium and 4.5g sugar in 100g dish (raw weight except for condiments). The cutoffs of nutrient contents according to the definition of "traffic lights" are listed in Table 2.

Table 2. The cutoffs of nutrient contents for dish nutrition evaluation[1]

Nutrient content	I	II	III
Fat (g/100g[2])	<8	8-20	>20
Sodium (mg/100g[2])	<500	500-1000	>1000
Sugar (g/100g[2])	<4.5	4.5-9	>9

[1] Green light for the dish represents all three indices within the range in the I column, red indicates at least 1 index within the range in the III column and yellow includes all the others.
[2] 100g refers to 100g edible portion of dish.

PN evaluation after meal consumption

PN evaluation after meal consumption is used to illustrate whether food intakes are inadequate, adequate or excessive.

A meal may consist of different dishes in varying portions. To obtain the actual intake of each chosen dish, nutritional values are multiplied by portions and non-discarded proportion that are input by participants on the ordering interface. Subsequently, to calculate the meal consumption, the consumptions of corresponding food groups, energy and nutrients from different dishes are summed up (Algorithm 2). The whole-day consumption can be further calculated according to self-reported contribution of three meals to total daily food intake.

Algorithm 2. Calculation of food groups, energy and nutrients for meal

$$meal = (dish[1], dish[2], \dots, dish[n])$$

$$Weight_{food\ group,meal} = \sum_{j \in meal} Weight_{food\ group,dish[j]} \times Portions[j] \times (1 - discarded\ proportion[j])$$

$$Energy_{meal} = \sum_{j \in meal} Energy_{dish[j]} \times Portions[j] \times (1 - discarded\ proportion[j])$$

$$Nutrient_{meal} = \sum_{j \in meal} Nutrient_{dish[j]} \times Portions[j] \times (1 - discarded\ proportion[j])$$

According to individual’s biological profile and total energy expenditure (TEE), the specific recommended intake of the individual will be determined. TEE can be calculated using resting energy expenditure (REE) multiplied by PAL [18]. REE is estimated by Schofield equation regarding age, sex and body weight [19]. PAL is categorized into 1.5 for light, 1.75 for moderate, and 2.0 for vigorous physical activity [20]. Based on Chinese Food Recommendation [21], TEE ranging from 1000 kcal/day to 3000 kcal/day can be divided to 11 ranks and nutrition needs for a balanced diet pattern vary with different TEE ranks. Taking self-reported contribution of three meals to total daily food intake into consideration, recommendations of food groups and nutrients per meal can be assessed according to Chinese Dietary Guidelines [22] and Chinese Dietary Reference Intakes (DRIs) [20], respectively.

By comparison with recommended intake, the PN evaluation regarding actual intake will be provided for participants. The key evaluation indices for meal consumption are described in Table 3, including fat, sodium, meat and vegetables. For each index, we assign a health score with a corresponding mark to illustrate the comparisons between the current consumption and the national dietary recommendations. For example, a mark of three plus (+++) for fat indicates that the percentage of energy intake from fat is far from recommendation. Evaluation of fat and sodium is given greater weight than others, with a score of 0-3, because these two dietary factors are more related to burden of NCDs in China [23]. Finally, an aggregate score of all the indices will be obtained, which represents the healthiness of whole meal consumption, 0 being the unhealthiest, and 10 being very healthy.

Table 3. The key evaluation indices for meal consumption

Evaluation index	Range	Health score	Mark
**Fat**: percentage of energy intake from fat, %	<20	2	—
	20-<30	3	Good
	30-<40	2	+
	40-<50	1	++
	50-<60	0.5	+++

	>60	0	++++
**Sodium**: actual intake, mg	<1000	3	Good
	1000-<1400	2	+
	1400-<1800	1	++
	1800-<2200	0.5	+++
	>2200	0	++++
**Meat**: actual/recommended intake ratio	<0.8	1	—
	0.8-<1.2	2	Good
	1.2-<1.4	1	+
	>1.4	0	++
**Vegetables**: actual/recommended intake ratio	<0.5	0	——
	0.5-<0.7	1	—
	>0.7	2	Good

Outcomes

In Phase I, dietary pattern, body weight or blood pressure optimizing is expected after the intervention. The primary outcome for the intervention effectiveness is dietary intakes. Anthropometric indicators including weight, body mass index (BMI), body composition and blood pressure are the secondary outcome. In Phase II, body metabolism normalization is expected after this period.

Quality control

The quality control team was established prior to this study. All the researchers involved in this study must attend the complete operation training, including study protocol and standard operating procedures of participants' data collection. In addition, both on-site and on-line review for data verification will be implemented, to ensure the quality and consistency of organizational operations, and stabilize food supply at the canteen.

Statistical analysis

The Mann-Kendall test will be implemented to examine the temporal trends of lunchtime food supply based on average "traffic light" score and animal/plant food ratio. For dietary record, we hypothesized that at least 5000 person-meal observations would be collected and analyzed over the follow-up period. Generalized linear mixed

models with intervention, time and 2-way interaction as fixed factors, will be used to examine the intervention effects on each outcome. A two-sided p value < 0.05 will be considered to indicate statistical significance.

Trial status

The recruitment for the trial was initiated in September 2022, but the field work of Phase I suspended in December 2022 due to the COVID-19 epidemic. Subsequent work is under arrangement at the time of submission. This protocol was completed before the research team had received any data.

Abbreviations

PN	Personalized nutrition
AI	Artificial intelligence
NCDs	Non-communicable diseases
RDs	Registered dietitians
DTRs	Registered dietetic technicians
RCTs	Randomized controlled trials
CNNS	China National Nutrition Survey
PAL	Physical activity level
TEE	Total energy expenditure
REE	Resting energy expenditure
DRIs	Dietary Reference Intakes
BMI	Body mass index

Reference

1. Collaborators GD. Health effects of dietary risks in 195 countries, 1990-2017: a systematic analysis for the Global Burden of Disease Study 2017. *Lancet*. May 11 2019;393(10184):1958-1972. doi:10.1016/s0140-6736(19)30041-8
2. Huang L, Wang Z, Wang H, et al. Nutrition transition and related health challenges over decades in China. Review. *EUROPEAN JOURNAL OF CLINICAL NUTRITION*. 2021 FEB 2021;75(2):247-252. doi:10.1038/s41430-020-0674-8
3. Zhai FY, Du SF, Wang ZH, Zhang JG, Du WW, Popkin BM. Dynamics of the Chinese diet and the role of urbanicity, 1991-2011. Review. *OBESITY REVIEWS*. 2014 JAN 2014;15:16-26. doi:10.1111/obr.12124
4. Gao C, Xu J, Liu Y, Yang Y. Nutrition Policy and Healthy China 2030 Building. Review. *EUROPEAN JOURNAL OF CLINICAL NUTRITION*. 2021 FEB 2021;75(2):238-246. doi:10.1038/s41430-020-00765-6
5. Yajie Z, Xiaoli W, Ya L, et al. An exploration of registered dietitian accreditation system development in China. *BMC Med Educ*. Dec 7 2022;22(1):846. doi:10.1186/s12909-022-03802-z
6. Bush CL, Blumberg JB, El-Sohemy A, et al. Toward the Definition of Personalized Nutrition: A Proposal by The American Nutrition Association. *J Am Coll Nutr*. Jan 2020;39(1):5-15. doi:10.1080/07315724.2019.1685332
7. Van Lippevelde W, Vangeel J, De Cock N, et al. Using a gamified monitoring app to change adolescents' snack intake: the development of the REWARD app and evaluation design. *BMC Public Health*. Aug 5 2016;16:725. doi:10.1186/s12889-016-3286-4
8. Zeevi D, Korem T, Zmora N, et al. Personalized Nutrition by Prediction of Glycemic Responses. *Cell*. Nov 19 2015;163(5):1079-1094. doi:10.1016/j.cell.2015.11.001
9. Oldenburg BF, Sallis JF, Ffrench ML, Owen N. Health promotion research and the diffusion and institutionalization of interventions. *Health Educ Res*. Feb 1999;14(1):121-30. doi:10.1093/her/14.1.121
10. Rickman AD, Williamson DA, Martin CK, et al. The CALERIE Study: design and methods of an innovative 25% caloric restriction intervention. *Contemp Clin Trials*. Nov 2011;32(6):874-81. doi:10.1016/j.cct.2011.07.002
11. Sarma H, D'Este C, Ahmed T, Bossert TJ, Banwell C. Developing a conceptual framework for implementation science to evaluate a nutrition intervention scaled-up in a real-world setting. *Public Health Nutr*. Apr 2021;24(S1):s7-s22. doi:10.1017/s1368980019004415
12. Luan C, Zhang M, Fan K, Devahastin S. Effective pretreatment technologies for fresh foods aimed for use in central kitchen processing. *J Sci Food Agric*. Jan 30 2021;101(2):347-363. doi:10.1002/jsfa.10602
13. Zhu Z, Zang J, Wang Z, et al. Dietary pattern and its seasonal characteristic in residents of Shanghai, 2012-2014. J. *Chinese Journal of Epidemiology*. 2018;39(7):880-885. doi:10.3760/cma.j.issn.0254-6450.2018.07.004
14. Song P, Zhang X, Li Y, et al. MetS Prevalence and Its Association with Dietary Patterns among Chinese Middle-Aged and Elderly Population: Results from a National Cross-Sectional Study. *Nutrients*. Dec 13 2022;14(24)doi:10.3390/nu14245301
15. Wu Y, Wang X, Gao F, Liao J, Zeng J, Fan L. Mobile nutrition and health management platform for perioperative recovery: an interdisciplinary research achievement using WeChat Applet. *Front Med (Lausanne)*. 2023;10:1201866. doi:10.3389/fmed.2023.1201866
16. YueXin Y. *China food composition tables standard edition*. 6 ed. 2018.

17. Zhu Z, Yang X, Fang Y, et al. Trends and Disparities of Energy Intake and Macronutrient Composition in China: A Series of National Surveys, 1982-2012. *Nutrients*. Jul 22 2020;12(8)doi:10.3390/nu12082168

18. Carlsohn A, Scharhag-Rosenberger F, Cassel M, Weber J, de Guzman Guzman A, Mayer F. Physical activity levels to estimate the energy requirement of adolescent athletes. *Pediatr Exerc Sci*. May 2011;23(2):261-9. doi:10.1123/pes.23.2.261

19. Schofield WN. Predicting basal metabolic rate, new standards and review of previous work. *Hum Nutr Clin Nutr*. 1985;39 Suppl 1:5-41.

20. Society CN. *Chinese Dietary Reference Intakes Handbook (2013)*. Beijing: China Standards Press; 2014.

21. Zhou J, Leepromrath S, Tian X, Zhou D. Dynamics of Chinese Diet Divergence from Chinese Food Pagoda and Its Association with Adiposity and Influential Factors: 2004-2011. *Int J Environ Res Public Health*. Jan 13 2020;17(2)doi:10.3390/ijerph17020507

22. Wang SS, Lay S, Yu HN, Shen SR. Dietary Guidelines for Chinese Residents (2016): comments and comparisons. *J Zhejiang Univ Sci B*. Sep 2016;17(9):649-56. doi:10.1631/jzus.B1600341

23. He Y, Li Y, Yang X, et al. The dietary transition and its association with cardiometabolic mortality among Chinese adults, 1982-2012: a cross-sectional population-based study. *Lancet Diabetes Endocrinol*. Jul 2019;7(7):540-548. doi:10.1016/s2213-8587(19)30152-4
