# Supplementary material for: Traffic Light Labels and Dietary Behavior Change: A Randomized Clinical Trial
Source: JAMA Netw Open. 2025 May 19;8(5):e2510894. doi: 10.1001/jamanetworkopen.2025.10894 (PMC12090027; doi:10.1001/jamanetworkopen.2025.10894)
Supplement: Supplement 2. — eFigure 1. The demonstration of the study canteen and the applet interfaces eFigure 2. The “Direction” of the applet interfaces eTable 1. Cutoffs of nutrients for “traffic light” classification on dishes eMethods 1. Calculation rules for “traffic light” classification on dishes eTable 2. The definition of covariates eMethods 2. Calculation rules for the average traffic light score eTable 3. Comparison of baseline characteristics between the participants included and excluded eTable 4. The distribution and quantity of green-coded, yellow-coded, and red-coded dishes in the daily and total menus eFigure 3. The temporal trend in the average traffic light score of the daily lunchtime menu eTable 5. Effects of the intervention on weekly changes of primary and secondary outcome eFigure 4. Effects of the intervention on primary and secondary outcomes across various subgroups at week 12 in model 1 [file jamanetwopen-e2510894-s002.pdf]

Supplementary Online Content

Liu H, Hu Z, Song Qi, Xu J, Mai S, Zhu Z. Traffic light labels and dietary behavior change: a randomized clinical trial. *JAMA Netw Open*. 2025;8(5):e2510894.
doi:10.1001/jamanetworkopen.2025.10894

eFigure 1. The demonstration of the study canteen and the applet interfaces

eFigure 2. The "Direction" of the applet interfaces

eTable 1. Cutoffs of nutrients for "traffic light" classification on dishes

eMethods 1. Calculation rules for "traffic light" classification on dishes

eTable 2. The definition of covariates

eMethods 2. Calculation rules for the average traffic light score

eTable 3. Comparison of baseline characteristics between the participants included and excluded

eTable 4. The distribution and quantity of green-coded, yellow-coded, and red-coded dishes in the daily and total menus

eFigure 3. The temporal trend in the average traffic light score of the daily lunchtime menu

eTable 5. Effects of the intervention on weekly changes of primary and secondary outcome

eFigure 4. Effects of the intervention on primary and secondary outcomes across various subgroups at week 12 in model 1

eReferences.

This supplementary material has been provided by the authors to give readers additional information about their work.

© 2025 Liu H et al. *JAMA Network Open*.

eFigure 1. The demonstration of the study canteen and the applet interfaces

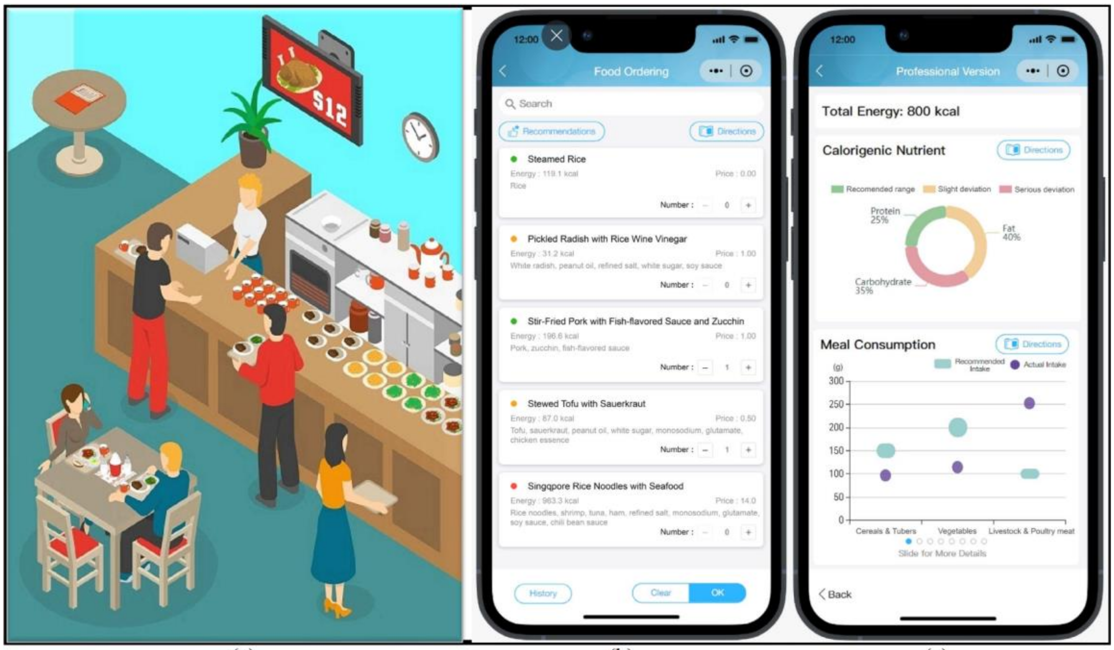

(a) (b) (c)

Note: eFigure 1a demonstrates the environment of the study canteen. eFigure 1b displays the ordering interface (i.e., the pre-meal dish nutrition evaluation ("traffic light" illustrations)) of the applet. eFigure 1c displays the nutrition report interface (i.e., post-meal personalized nutrition report) of the applet.

© 2025 Liu H et al. *JAMA Network Open*.

eFigure 2. The “Direction” of the applet interfaces

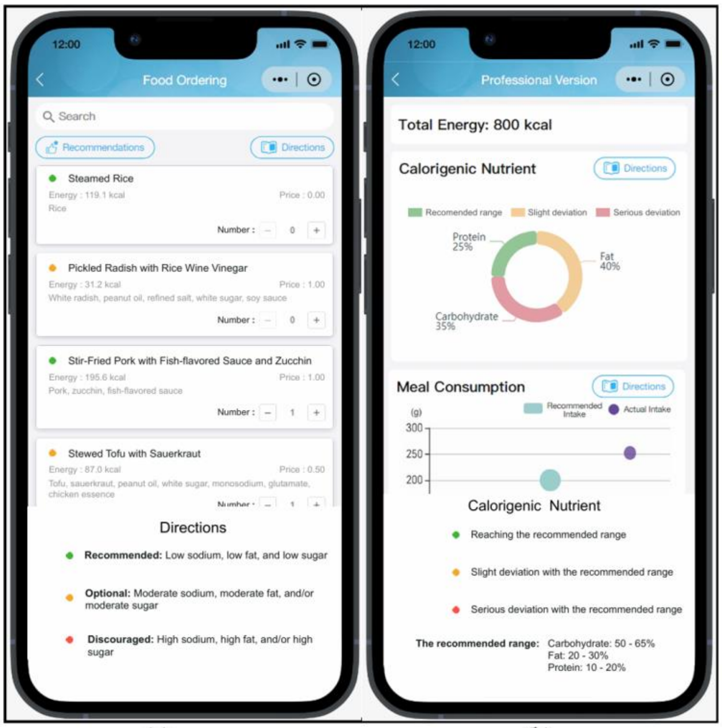

(a) (b)

Note: eFigure 2a demonstrates the “Direction” of the ordering interface. eFigure 2b displays the “Direction” of the nutrition report interface.

eTable 1. Cutoffs of nutrients for "traffic light" classification on dishes [a]

Nutrients, per 100 g [b]	I	II	III
Fat, g	<8	8–20	>20
Sodium, mg	<500	500–1000	>1000
Sugar, g	<4.5	4.5–9.0	>9.0

Note: Note: [a] Green for the dish represents all three items within the range in the I column, red indicates at least 1 item within the range in the III column, and yellow includes all the others. [b] 100 g refers to 100 g edible portion of dish.

© 2025 Liu H et al. *JAMA Network Open*.

eMethods 1. Calculation rules for "traffic light" classification on dishes

The cut-off values for traffic lights were determined based on dietary recommendations [1] and the average intake of the Chinese population [2]. Total daily food intake was adjusted by excluding staple foods, fruits, and milk, yielding a food pool of 650g/day [1]. The initial thresholds for added sugar, fat, and sodium per 100g of food were firstly calculated by scaling the dietary recommendations and the average intakes to this adjusted total daily food intake, respectively. However, since the content of added sugar, fat, and sodium in most dishes offered in the canteen exceeded the first threshold (based on dietary recommendations) and clustered around the second threshold (based on average intake), the distribution of green-, yellow-, and red-coded dishes in both daily and total menus lacked sufficient differentiation. This limited the effectiveness of the traffic light labels in encouraging participants to choose healthier foods. To ensure practical utility, the final thresholds were appropriately adjusted and rounded to ensure a more balanced distribution and quantity of green-, yellow-, and red-coded dishes (green: 40%, yellow: 45%, red: 15%) in both daily and total menus. Ultimately, the content of added sugar, fat, and sodium in 100g of each dish (raw weight, excluding condiments) was compared against these finalized thresholds to assign the corresponding color codes. This approach could balance public health targets with real-world feasibility.

eTable 2. The definition of covariates

Covariates	Categories	Definition
Smoking status	Non-smoker	
	Ex-smoker	
	Current Smoker	
Alcohol consumption	Lifetime abstainers	Had never consumed a drink
	No-heavy drinkers	Drinking alcohol approximately 1 to 6 times per year
	Heavy drinkers	Drinking alcohol more than 6 times per year
Physical activity	Low	
	Moderate	Self-rating on daily physical activity
	Vigorous	
Intentional physical exercise	Yes	
	No	
BMI [a]	Underweight	<18.5 kg/m^2
	Normal weight	18.5 to 23.9 kg/m^2
	Overweight	24.0 to 27.9 kg/m^2
	Obesity	>= 28.0 kg/m^2
Nutrition literacy		The responses to five questions regarding nutrition knowledge were aggregated, with higher scores denoting greater nutrition literacy. 1. "At the basis of a balanced dietary structure, the dishes containing excessive cooking oil and salt can be consumed". The response options ranging from “yes” (coded as 1) to “no” (coded as 0); 2. "Do you think it is important to manage your daily diet?". The response options include "important" (coded as 1), "less important" (coded as 0.5), and "unnecessary" and "completely unnecessary" (coded as 0); 3. "Do you pay attention to the energy and nutrients you consume in your diet?". The response options ranging from "yes" (coded as 1) to "no" (coded as 0); 4. "Do you have the ability to assess whether foods consumption and nutrients intake of a meal were rational and adequate?" The response options include "having the ability" (coded as 1), "partly having the ability" (coded as 0.5), and "having no the ability" (coded as 0); 5. "When you order meals, do you pay attention to whether the dishes are healthy?". The response options include "frequently" (coded as 1), "occasionally" (coded as 0.5), and "no" (coded as 0).
Demand for dietary guidance		The responses to three questions regarding demand for dietary guidance were aggregated, with higher scores denoting greater demand for dietary guidance. 1. "how do you think about your health condition?". The response options ranging from “very good” (coded as 0) to “very bad” (coded as 1); 2. "how do you think about your weight". The response options include "normal" (coded as 0) and "fat or thin" (coded as 1); 3. "Do you wish to receive dietary consultation services?". The response options include "need" (coded as 1) and "don't matter" (coded as 0).

Note: [a] BMI was calculated by dividing weight (in kilograms) by height (in meters) squared and categorized by the reference standard for the Chinese population.

© 2025 Liu H et al. *JAMA Network Open*.

eMethods 2. Calculation rules for the average traffic light score

The red-coded dish was defined as 3 point, yellow-coded dish was defined as 2 point, and green-coded dish was defined as 1 point.

$$The\ average\ traffic\ light\ score = \frac{3*The\ number\ of\ red-coded\ dishes + 2*The\ number\ of\ yellow-coded\ dishes + 1*The\ number\ of\ green-coded\ dishes}{The\ number\ of\ dishes}$$

© 2025 Liu H et al. *JAMA Network Open*.

eTable 3. Comparison of baseline characteristics between the participants included and excluded

	ALL<br>n = 177	Participants included<br>n = 153	Participants excluded<br>n = 24	P value
Age, mean (SD)	33.1 (7.8)	32.7 (7.5)	35.7 (9.2)	0.08
Sex, No. %				
Female	101 (57.1)	97 (63.4)	4 (16.7)	<0.001
Male	76 (42.9)	56 (36.6)	20 (83.3)	
Smoking status, No. %				
Non-smoker	150 (84.7)	136 (88.9)	14 (58.3)	<0.001
Ex-smoker	14 (7.9)	11 (7.2)	3 (12.5)	
current Smoker	13 (7.3)	6 (3.9)	7 (29.2)	
Alcohol consumption, No. %				
Lifetime abstainer	72 (40.7)	67 (43.8)	5 (20.8)	0.001
Non-heavy drinkers	77 (43.5)	68 (44.4)	9 (37.5)	
Heavy drinkers	28 (15.8)	18 (11.8)	10 (41.7)	
Physical activity, No. %				
Low	154 (87.0)	134 (87.6)	20 (83.3)	0.31
Moderate	21 (11.9)	18 (11.8)	3 (12.5)	
High	2 (1.1)	1 (0.7)	1 (4.2)	
Entended Exercise, No. %				
No	135 (76.3)	118 (77.1)	17 (70.8)	0.68
Yes	42 (23.7)	35 (22.9)	7 (29.2)	
Enrollment period, No. %				
September	23 (13.0)	23 (15.0)	0 (0.0)	0.12
October	101 (57.1)	86 (56.2)	15 (62.5)	
November	53 (29.9)	44 (28.8)	9 (37.5)	
BMI, No. %				
Underweight	7 (4.0)	6 (3.9)	1 (4.5)	0.03
Normal	104 (59.4)	96 (62.7)	8 (36.4)	
Overweight	48 (27.4)	36 (23.5)	12 (54.5)	
Obesity	16 (9.1)	15 (9.8)	1 (4.5)	
Missing, No. %	2 (1.1)	0 (0.0)	2 (8.3)	

Note: Independent samples t-tests was conducted for continuous variables and chi-square tests was conducted for categorical variables

© 2025 Liu H et al. *JAMA Network Open*.

eTable 4. The distribution and quantity of green-coded, yellow-coded, and red-coded dishes in the daily and total menus

	Dish			
	Green-coded	Yellow-coded	Red-coded	Total
Total number, n	78	121	42	241
Daily number, median (IQR)	6 (4.75-6)	7 (6-8)	2 (1-2)	14 (14-14)

© 2025 Liu H et al. *JAMA Network Open*.

eFigure 3. The temporal trend in the average traffic light score of the daily lunchtime menu

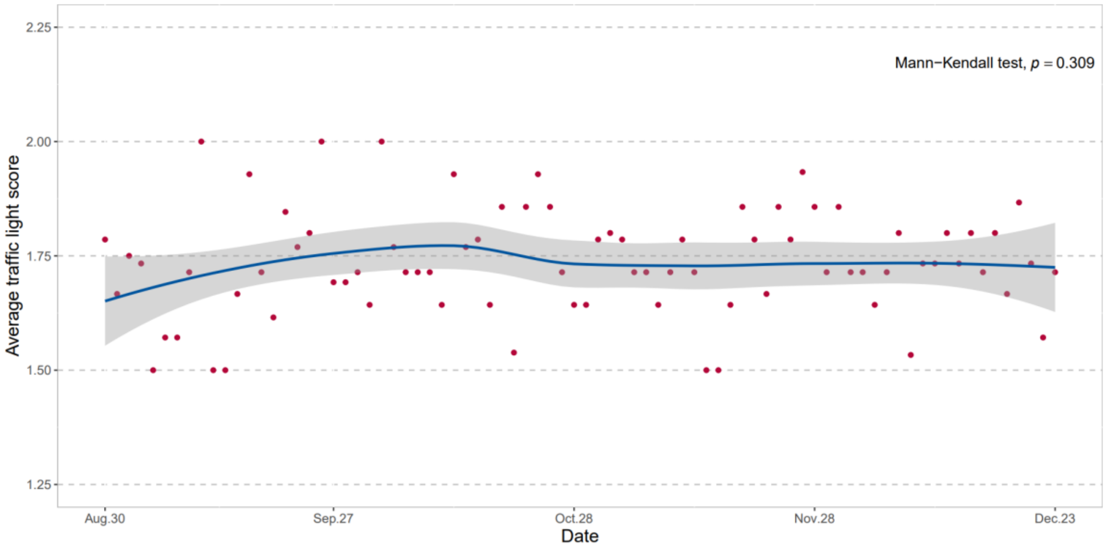

Note: Red dots represented daily average traffic light score, while the smoothing curve displayed the trend of this score over time

© 2025 Liu H et al. *JAMA Network Open*.

eTable 5. Effects of the intervention on weekly changes of primary and secondary outcome

	Time × Group [a]					
	Model 1[b]			Model 2 [c]		
	β [d] (95% CI)	OR [d] (95% CI)	*p*	β [d] (95% CI)	OR [d] (95% CI)	*p*
Dietary intake						
Added sugar, g/meal	-0.03 (-0.09, 0.04)		0.44	-0.03 (-0.02, 0.04)		0.44
Fat, g/meal	-0.05 (-0.48, 0.38)		0.83	-0.03 (-0.46, 0.40)		0.89
Sodium, mg/meal	-14.68 (-49.98, 20.62)		0.41	-15.01 (-49.45, 19.43)		0.39
Average traffic light score	-0.005 (-0.013, 0.003)		0.19	-0.005 (-0.012, 0.003)		0.22
The numbers of dishes						
Green-coded		1.12 (0.96, 1.30)	0.15		1.01 (0.99, 1.03)	0.26
Yellow-coded		1.003 (0.99, 1.02)	0.74		1.00 (0.99, 1.02)	0.84
Red-coded		0.98 (0.94, 1.03)	0.36		0.98 (0.94, 1.03)	0.37

Note: Note: [a] The significant interaction item (Time × Group) indicated overall trend differences between the both groups; [b] Model 1 adjusted for the baseline value of average traffic light score consumed and enrollment sequence; [c] Model 2 adjusted for the baseline value of average traffic light score consumed, enrollment sequence, age, sex, smoking status, alcohol consumption, physical activity level, intentional physical exercise, BMI, nutrition literacy, and demand for dietary guidance; [d] represented average weekly change in dietary choice or consumption; CI, confidence interval.

© 2025 Liu H et al. *JAMA Network Open*.

eFigure 4. Effects of the intervention on primary and secondary outcomes across various subgroups at week 12 in model 1

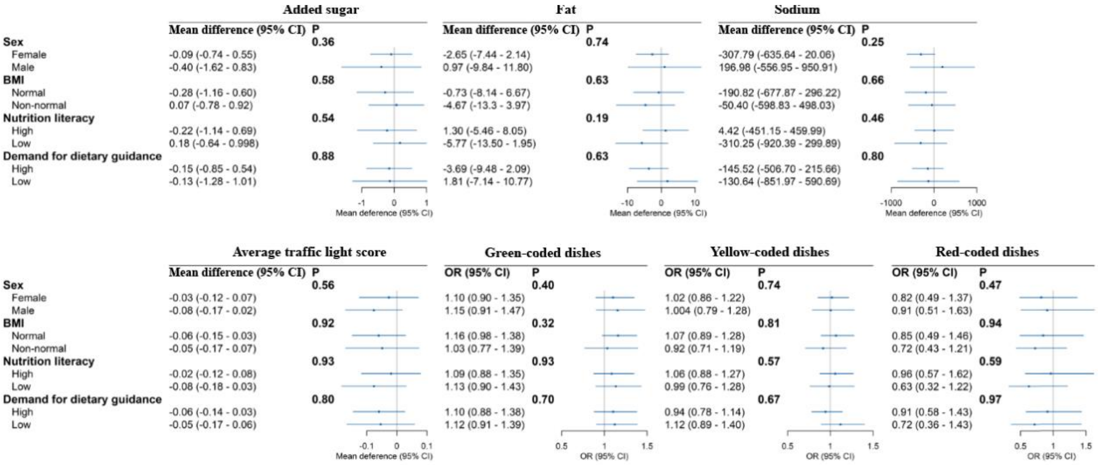

Note: Model 1 adjusted for the baseline value of average traffic light score consumed and enrollment sequence; BMI was reclassified into normal or non-normal (underweight, overweight, and obesity). Both nutrition literacy and demand for dietary guidance were divided into low (scores below the median) or high (scores at or above the median); CI, confidence interval.

© 2025 Liu H et al. *JAMA Network Open*.

eReferences.

1. Chinese Nutrition Society. Dietary reference intakes for China (2023). People's Medical Publishing House; 2023.
2. Zhu Z, Yang X, Fang Y, et al. Trends and Disparities of Energy Intake and Macronutrient Composition in China: A Series of National Surveys, 1982-2012. Nutrients. Jul 22 2020;12(8)doi:10.3390/nu12082168

© 2025 Liu H et al. *JAMA Network Open*.
