# Supplementary material for: Traffic Light Labels and Dietary Behavior Change: A Randomized Clinical Trial
Source: JAMA Netw Open. 2025 May 19;8(5):e2510894. doi: 10.1001/jamanetworkopen.2025.10894 (PMC12090027; doi:10.1001/jamanetworkopen.2025.10894)
Supplement: Supplement 3. — Data Sharing Statement [file jamanetwopen-e2510894-s003.pdf]

Data Sharing Statement

Liu. Traffic Light Labels and Dietary Behavior Change. *JAMA Netw Open*. Published May 19, 2025. doi:10.1001/jamanetworkopen.2025.10894

Data
Additional Information: Trial Registration: Chinese Clinical Trial Registry: ChiCTR2100051771
Data available: Yes
Data types: Deidentified participant data
How to access data: Send request email to Dr. Zhu (zhuzhenni@scdc.sh.cn)
When available: With publication

Supporting Documents
Document types: None

Additional Information
Who can access the data: Researchers whose proposed use of the data has been approved
Types of analyses: Only for research
Mechanisms of data availability: With investigator support
